# Microproteins as a Hidden Player in Breast Cancer: From Molecular Insights to Therapeutic Potential

**DOI:** 10.1002/cbf.70305

**Published:** 2026-09-11

**Authors:** Qianyi Dai, Kimberly Seaman, Xin Song, Amel Sassi, Xiaolong Yang, Lidan You

**Affiliations:** ^1^ Department of Mechanical and Materials Engineering Queen's University Kingston Ontario Canada; ^2^ Department of Mechanical and Industrial Engineering University of Toronto Toronto Ontario Canada; ^3^ Institute of Biomedical Engineering University of Toronto Toronto Ontario Canada; ^4^ Department of Pathology and Molecular Medicine Queen's University Kingston Ontario Canada

**Keywords:** breast cancer (BC), cancer diagnosis, cancer prognosis, cancer therapy, micropeptide, microproteins, small open reading frame (smORF)

## Abstract

Breast cancer continues to pose a public health problem and is the second leading cause of cancer‐related mortality among women globally. Despite advances in our understanding of cancer biology and the development of treatment strategies, the therapeutic response is not often satisfactory as treatment efficacy is limited by severe side effects and drug resistance. Therefore, there is an urgent need for innovative approaches to improve diagnosis, prognosis, and treatment of breast cancer. Recent advances in bioinformatics and proteomics have enabled the discovery of a novel class of small proteins encoded from the small open reading frames, which were previously thought to be non‐coding. Emerging evidence has indicated that these microproteins play a pivotal role in modulating breast cancer progression. This article reviews the current understanding of microproteins, highlights the microproteins identified to date as key regulators in breast cancer, and explores their potential clinical applications in improving breast cancer management.

## Summary

- Microproteins encoded by smORFs have recently been found to play a role in the regulation of breast cancer proliferation, metastasis, and chemoresistance, even though they were previously regarded as non‐coding products.
- Thirteen breast cancer–associated microproteins are catalogued, with tumour‐promoting and tumour‐suppressive roles distinguished.
- Three microproteins affect Wnt/β‐catenin signalling in opposing ways, depending on the cellular context and the subcellular location of β‐catenin.
- Preclinical models show direct administration of tumour‐suppressive microproteins (ASRPS, miPEP205, and CIP2A‐BP) reduces metastasis and prolongs survival.
- Clinical translation is constrained by proteolytic instability, sub‐threshold molecular size and renal clearance, and immunogenicity—defining the engineering agenda for microprotein therapeutics.
- Microproteins, including MRP, MTLN, ASRPS, and HCP5‐132aa, show early promise as diagnostic and prognostic biomarkers, particularly in TNBC.

**Abbreviations**
aa: amino acid (to define microprotein length)
ADR: adriamycin
altORFs: alternative ORFs
ASRPS: a small regulatory peptide of STAT3
BC: breast cancer
BCSCs: breast cancer stem cells
CASIMO1: Cancer‐Associated Small Integral Membrane Open Reading Frame 1
circRNA: circular RNA
CSCs: cancer stem cells
ECM: extracellular matrix
EGFR: epidermal growth factor receptor
ER: endoplasmic reticulum
HCP5: human leucocyte antigen complex P5
lncRNA: long non‐coding RNA
LY6E‐DT: LY6E divergent transcript
MAGI2‐AS3: MAGI2 antisense RNA 3
MRP: metastatic‐related protein
MTLN: mitoregulin
mTOR: mammalian target of rapamycin
mTORC1: the mTOR complex 1
ncRNA: non‐coding RNA
P27KIP1: cyclin‐dependent kinase inhibitor 1B
PPIs: protein–protein interactions
PROTACs: proteolysis‐targeting chimeras
ROS: reactive oxygen species
SERCA: sarco/endoplasmic reticulum Ca2+‐ATPase
SLN: sarcolipin
smORFs: small open reading frames
sORFs: short ORFs
SPAR: small regulatory polypeptide of amino acid response
SQLE: squalene epoxidase
STAT3: signal transducer and activator of transcription 3
TGF‐β: transforming growth factor beta
TNBC: triple‐negative breast cancer
TRPC5OS: TRPC5 opposite strand
TRPC5‐AS1: TRPC5 antisense RNA 1
VEGF: vascular endothelial growth factor

## 1 Introduction

Cancer remains a major health crisis in the 21st century, responsible for nearly one in six deaths worldwide [1]. Breast cancer (BC) is the most commonly diagnosed malignancy among women worldwide and the second most common cancer overall [1, 2]. In 2024, approximately one in eight women in the US were diagnosed with invasive BC, with a 2.3% mortality rate, according to the American Cancer Society [3]. These statistics highlight the urgent need to identify and characterise novel biomarkers and oncogenic targets to enable more effective, targeted therapies for BC.

Advances in next‐generation sequencing techniques have uncovered a new class of proteins produced by the translation of small open reading frames (smORFs) [4]. These proteins are typically shorter than 300 base pairs, and are mostly found on non‐coding RNAs (ncRNAs), which comprise up to 98% of the transcriptome in humans [4]. These proteins are referred to as microproteins, micropeptides, or small ORF‐encoded peptides [5, 6]. Their inherently small size is the main reason they were historically excluded by most ORF prediction algorithms, which apply a threshold of 300 bp [7]. Although only a small portion of them have been identified, emerging research shows their important functions as modulators of cellular processes such as RNA processing, DNA repair, metabolism regulation and regeneration [8, 9, 10, 11]. They also interface with signalling cascades, metabolic pathways, and epigenetic machinery to fine‐tune biological responses [12]. These discoveries have significantly expanded our understanding of the regulatory complexity of the genome, with profound implications for both fundamental research and clinical applications. While microproteins are conventionally defined as proteins ≤ 100 amino acids (aa), we adapt a broader working definition (up to 171 aa) to accommodate all proteins of interest discussed herein, consistent with evolving usage in the field.

A growing body of research has revealed that a subset of recently characterised microproteins are directly linked to cancer and their behaviours [13, 14]. For example, Zheng et al. proposed that a 73 aa microprotein encoded by circPPP1R12A promotes colon cancer proliferation via the Hippo‐Yap signalling pathway [15]. More recently, a long non‐coding RNA (lncRNA) that links to the Y chromosome named LINC00278 has been reported to encode a microprotein named YY1BM with tumour suppressor effects. Intratumoural injection of LINC00278 resulted in tumour mass elimination in xenograft models [16]. Collectively, these studies demonstrate the critical role of microproteins as cancer regulators.

There are four main clinical BC subgroups: luminal A, luminal B, human epidermal growth factor receptor 2positive, and triple‐negative breast cancer (TNBC) [17]. Each of these subtypes presents distinct challenges in prognosis and treatment, largely due to their molecular heterogeneity and variable therapeutic responses. In this context, microproteins encoded by smORFs are emerging as promising candidates for cancer research. Their tissue‐specific expression, dynamic regulation during tumour progression, and involvement in processes such as immune evasion and metastasis highlight their potential to serve as novel biomarkers or therapeutic targets in addressing the molecular heterogeneity of BC [18, 19, 20]. Despite growing interest in this field, a comprehensive synthesis of current knowledge on microproteins in BC is lacking. This review aims to present the latest understanding of microproteins in the context of BC, including the identified microproteins associated with BC, and explores their clinical implications and future translational opportunities.

## 2 Biology of Microproteins

smORFs that encode microproteins can be found in various genomic contexts, including regions previously thought to be non‐coding, such as 5′ and 3′ untranslated regions on known protein‐coding genes, namely upstream and downstream open reading frames [13]. Alternatively, smORFs can also originate from the main ORF, intergenic regions and pseudogenes. In the mitochondria, smORFs are found in mitochondrial DNA. Within the cytoplasm, smORFs are scattered across various RNA transcripts, such as circular RNA (circRNA), lncRNA, and pri‐microRNA [13, 21]. Some literature also refers to smORFs as short ORFs (sORFs), which are often used to distinguish them from alternative ORFs (altORFs). By definition, sORFs are found within transcripts previously considered non‐coding (microRNA, lncRNA, and circRNA), while altORFs are located within regions that overlap with annotated coding genes [22].

**FIGURE 1:**
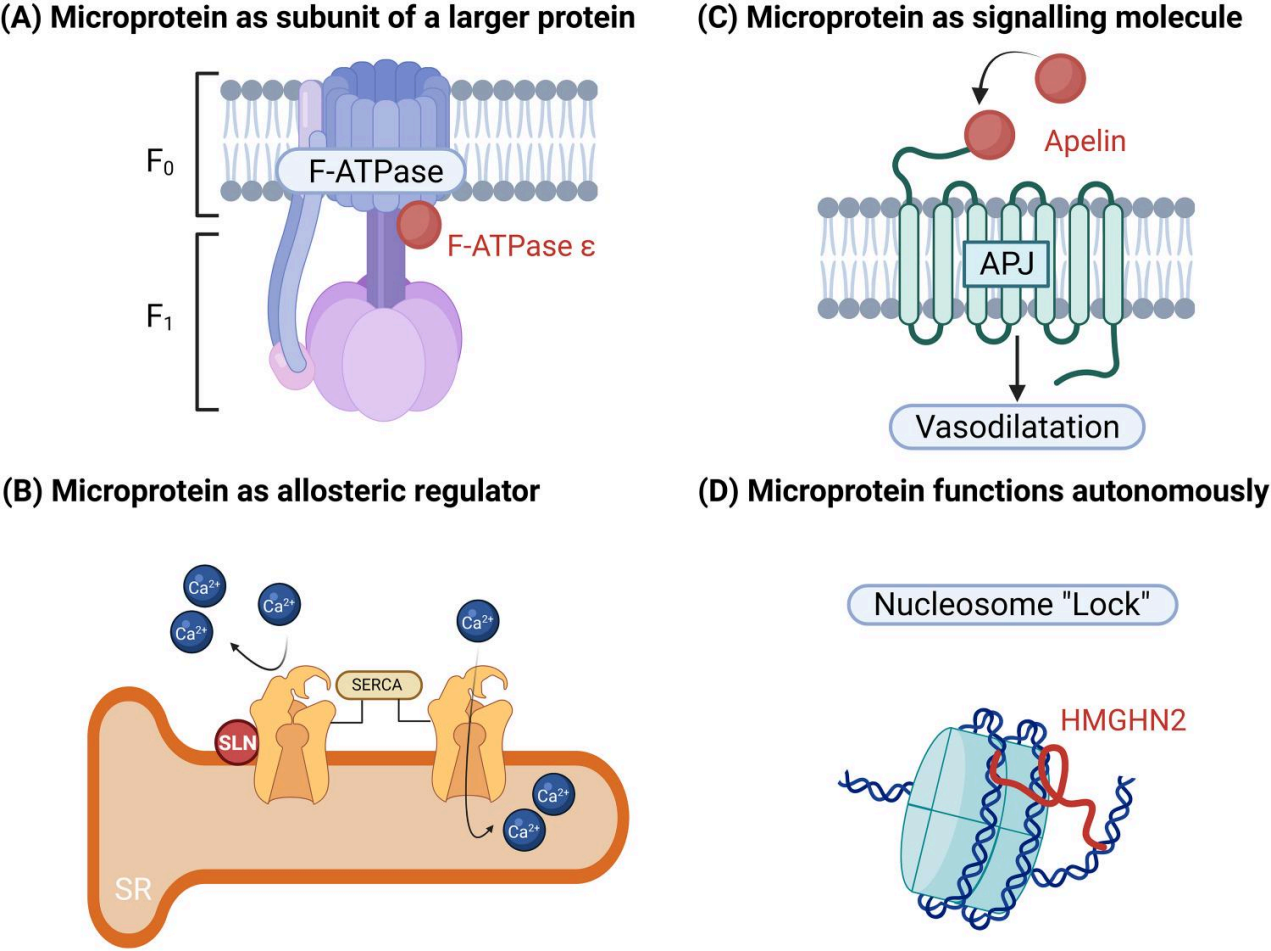
Molecular mechanism of microproteins (created with BioRender.com).

Microproteins encoded by smORFs play important roles at the molecular, structural, and/or mechanistic level, contributing to diverse cellular and organismal functions [23, 24]. Many act as critical subunits within larger protein complexes, where they stabilise interactions or modulate activity (Figure 1A). For instance, the mammalian mitochondrial F‐ATPase epsilon functions with a 50–65 aa subunit acting as an assembly or stabilising factor within F0 and F1 subcomplexes [24, 25]. Some microproteins function as allosteric regulators, inducing conformational changes that alter the activity of target proteins (Figure 1B). For example, a 31‐aa‐long microprotein, sarcolipin (SLN), is a regulator of sarco/endoplasmic reticulum Ca^2+^‐ATPase (SERCA) pump in muscle cells [26]. By binding to the E2 conformation of SERCA 1a, SLN hinders the transition of SERCA back to its E1 conformation, which impairs the necessary calcium uptake [26]. Microproteins can also serve as signalling molecules (Figure 1C), such as the secreted microprotein Apelin, which activates apelin receptors to regulate cardiovascular homeostasis by inducing endothelium‐ and nitric oxide‐dependent vasodilatation [27]. Finally, certain microproteins operate autonomously to perform essential tasks (Figure 1D). For example, HMGN2, a 90 aa microprotein, interacts with the histone H2A‐H2B dimer and the DNA at the entry and exit sites of the nucleosome, “locking” the histone core and DNA together [28]. Yet, the role of microproteins in chromatin packaging, condensation, and the regulation of DNA‐templated processes, such as transcription and replication, remains poorly understood. This functional versatility enables microproteins to act as precise modulators of cellular homeostasis, capable of influencing processes ranging from DNA repair to energy metabolism.

## 3 Functional Microproteins in BC

While the functions and underlying mechanisms of microproteins are complex and have not yet been fully elucidated, emerging studies have identified several microproteins with distinct expression profiles associated with BC, including roles in progression, proliferation, migration, and chemoresistance (Table 1). In the following section, we categorise these functional microproteins based on their roles as BC promoters and suppressors. It should be noted that precise functional mechanisms for BC microproteins presented below have not been characterised. Their connections to the functional categories in Section II and Figure 1 are proposed as potential mechanisms in Table 1. However, it should be noted that the mechanisms are still largely inferred and require experimental validation.

**TABLE 1:** Summary of microproteins associated with BC. The letters under Potential Mechanisms correspond to the letters presented for each functional category in Figure 1. Microproteins that fall under the expanded definition in this review (larger than 100aa but smaller or equal to 171aa) are indicated with an asterisk.

Microprotein	Size (aa)	Expression pattern	Potential mechanism (corresponds to Figure 1)	Role(s) in BC	Reference
Tumour promoters					
MTLN	56	Highly expressed in BC cells	C	Supports ER stress‐adaptation response that retains cancer proliferation	[29]
CAPG‐171aa*	171	Encoded by circCAPG that is highly expressed in TNBC tumour samples	C	Promotes tumour growth and metastasis	[30]
MRP*	153	High expression in metastatic cancer tissue samples	B	Promotes migration and metastasis	[31]
TRPC5OS*	111	Highly expressed in BC cells	C	Promotes cancer proliferation and cell cycle progression	[32]
CASIMO1	83	Highly expressed in ER/PR+ breast tumour samples	C	Retains cancer proliferation and migration, modulates lipid droplet accumulation	[33]
LINC00511‐133aa*	133	Encoded by LINC00511 that is highly expressed in metastatic BC cells	C	Promotes cancer cell metastasis and stemness	[34]
HCP5‐132aa*	132	High expression in TNBC, ADR‐resistant BC cells and tumour samples	C	Increases tumour growth and chemoresistance to ADR	[35, 36]
Tumour suppressors					
SPAR	48	Low expression in BC cells and tumour samples	C	Represses cancer proliferation	[37]
miPEP205	49	Significantly lower expression in HER2+ and TNBC breast tumour samples	C	Suppresses tumour growth and lung metastasis	[38]
ASRPS	60	Reduced expression level in TNBC	B	Reduces tumour angiogenesis and cell growth	[39]
MAGI2‐AS3‐ORF5	Undetermined	Low expression in invasive BC	B	Restrains tumour viability, proliferation, and migration	[40]
CIP2A‐BP	52	Low expression in TNBC models	D	Inhibits cancer cell invasion and metastasis	[18]

### 3.1 Microproteins as Promoters of Tumour Growth and Metastasis

Due to their rapid growth and constant need for new proteins, cancer cells are inevitably subjected to endoplasmic reticulum (ER) stress [40]. To manage ER stress during repeated mitosis, cells activate multiple repair pathways, with one critical aspect involving the communication between the ER and mitochondria. This crosstalk occurs at mitochondria‐associated ER membranes, which are vital for regulating apoptosis, autophagy, and inflammasome activation [41]. Mitoregulin (**MTLN**), a microprotein encoded by LINC00116, has recently been reported not only to control mitochondrial functions in skeletal muscle cells and adipocytes [42, 43] but also to contribute to the precise control of ER‐mitochondria interaction in ER‐stress‐exposed BC cells. A deficiency in MTLN disrupts this communication, leading to diminished expression of genes responsible for the ER stress response [29].

More often, some microproteins promote not only the growth but also the migration of cancer cells. For instance, **CAPG‐171aa** is a microprotein encoded by the circRNA, circCAPG. It was found highly expressed in TNBC and strongly associated with advanced clinical stages and poorer prognosis. More importantly, studies have indicated that CAPG‐171aa plays a crucial role in tumour growth and metastasis by interacting with serine/threonine kinase 38 [30]. An in vitro mechanistic study revealed that this interaction prevents the ubiquitination and degradation of mitogen‐activated protein kinase kinase 2, leading to activation of the Mitogen‐Activated Protein Kinase/Extracellular Signal‐Regulated Kinase Kinases 1 and 2 signalling pathway and promoting further proliferation and metastatic spread of TNBC cells [30].

Similarly, Liu et al. found for the first time that the LY6E divergent transcript (LY6E‐DT) encodes a conserved 153 aa protein in BC tissues, which they named “Metastatic‐Related Protein” (**MRP**) [31]. Both LY6E‐DT and MRP contribute to BC progression by different molecular mechanisms, but LY6E‐DT RNA and protein levels were increased in highly metastatic cancer tissues compared with low‐metastatic ones, indicating their active involvement in tumour metastasis [31]. The later mass spectrometry results suggest that MRP mainly interacts with hnRNPC, an RNA‐binding protein that regulates RNA splicing and processing [31]. This interaction enhances the stability and expression of epidermal growth factor receptor (EGFR), subsequently activating EGFR signalling that promotes BC metastasis [31].

A 111 aa microprotein, **TRPC5OS**, that translated from TRPC5 opposite strand (TRPC5OS), or TRPC5 antisense RNA 1 (TRPC5‐AS1) has been found to play a role in BC growth and progression [32]. Elevated levels of TRPC5OS have been observed in BC tissues compared to their normal counterparts. Identified as an oncogenic molecule, TRPC5OS overexpression not only promotes BC cell proliferation and accelerates cell cycle progression in vitro, but also enhances tumour growth in xenograft models in vivo [32]. Mechanistic studies using immunoprecipitation and MS analyses have shown that TRPC5OS interacts with members of the glycolytic pathway, notably alpha‐enolase [32]. Given that increased glucose metabolism is essential for the sustained unlimited growth of tumour cells [44], the positive correlation between TRPC5OS expression, tumour size, and tumour grade by affecting ENO1 protein levels and downstream PI3K/Akt activity, as confirmed by measuring glucose uptake—these highlights its potential as both a prognostic marker and a therapeutic target [32].

Furthermore, some microproteins regulate BC cell motility. Polycarpou‐Schwarz et al. identified and characterised a new gene, Cancer‐Associated Small Integral Membrane Open Reading Frame 1 (**CASIMO1**), which is predominantly overexpressed in Luminal A breast tumours [33]. The knockdown of CASIMO1 in the three BC cancer cell lines (MCF7, KPL1, and T47D) disrupted the organisation of the actin cytoskeleton, leading to reduced cell motility and an arrest in the G0/G1 phase of the cell cycle. Moreover, CASIMO1 was found to interact with squalene epoxidase (SQLE) [33], a key enzyme coding gene in cholesterol synthesis and a known oncogene in BC. High levels of CASIMO1 contribute to SQLE protein accumulation and increase lipid droplet clustering, which meets the demand of the rapid proliferation of cancer cells [33]. These findings establish CASIMO1 as the first functional microprotein that plays a role in carcinogenesis and is implicated in cell lipid homeostasis.

Cancer stem cells (CSCs), also known as tumour‐initiating cells, represent a distinct subpopulation within tumours characterised by their abilities for self‐renewal, generation of the heterogeneous lineages that comprise the tumour, and long‐term tumour propagation upon serial transplantation [45]. CSCs are widely recognised as the root drivers of tumour progression, invasion, metastasis, resistance to chemotherapy, and relapse [46]. Among the pathways implicated in CSC regulation, the Wnt/β‐catenin signalling pathway has been identified as a key contributor to the maintenance and function of BC stem cells (BCSCs) [47]. Tan et al. revealed that **LINC00511‐133aa**, a microprotein encoded by the lncRNA LINC00511, upregulates stemness markers Oct4, Nanog, and SOX2. Sphere‐formation assays and Western blot analyses further support the role of LINC00511‐133aa in activating the Wnt/β‐catenin pathway and promoting stemness in TNBC cell lines (MDA‐MB‐231, MDA‐MB‐468, and HCC‐1937 cells) [34].

Apart from cell growth, some microproteins support cancer cells by enhancing their resistance to conventional therapeutic approaches. The lncRNA human leucocyte antigen complex P5 (HCP5) encodes a 132 aa microprotein, referred to as **HCP5‐132aa**. This microprotein is significantly upregulated in TNBC cell lines (MDA‐MB‐231 and MDA‐MB‐468) and primary tumour tissues compared to normal tissues [35]. HCP5‐132aa plays a pivotal role in driving TNBC malignancy by modulating the ferroptosis pathway that induces programmed cell death characterised by the iron‐dependent accumulation of lipid reactive oxygen species (ROS) [35]. Knockdown of the HCP5‐132aa ORF alone was sufficient to trigger ferroptosis, as evidenced by elevated ROS levels, reduced mitochondrial cristae, and enhanced membrane density [35]. However, the precise molecular mechanism by which HCP5‐132aa induces ferroptosis remains unclear. In addition to its role in TNBC, HCP5‐132aa expression is elevated in Adriamycin (ADR)‐resistant MCF‐7/ADR cells compared to their parental MCF‐7. ADR (also known as doxorubicin) is a widely used chemotherapeutic agent in BC treatment [36]. Notably, silencing HCP5‐132aa increased the sensitivity of these cells to ADR, as evidenced by enhanced growth inhibition following treatment [36].

### 3.2 Microproteins as Suppressors of Tumour Growth

Contrastingly, some microproteins repress cancer cell growth and/or metastasis. The mammalian target of rapamycin (mTOR), a serine/threonine kinase integral to the mTOR complex 1 (mTORC1), serves as a central hub for cell growth by connecting nutrient signals with metabolic processes, upon amino acid response. In addition, the cyclin‐dependent kinase inhibitor 1B (P27KIP1) has been identified as a noncanonical inhibitor of mTOR signalling in mouse embryo fibroblasts, and its dysregulation is common in cancer cells [48]. Research by Matsumoto et al. revealed that lncRNA LINC00961 encodes a functional microprotein known as a small regulatory polypeptide of amino acid response (**SPAR**). Notably, SPAR localises to lysosomes in several cell types, including mouse muscle cells, HEK293T cells, HeLa cells, and prostate cancer PC3 cells, in which it interacts with V‐ATPase, inhibits mTORC1 signalling activity and suppresses muscle regeneration [49]. Furthermore, glutamine is one of the most abundant amino acids in cells and an important nutrient for mTOR signalling activation [50], thereby sustaining the proliferation of both non‐cancerous and cancerous cells. This dependency has stimulated interest in targeting glutamine metabolism for anti‐cancer treatment. In this context, the microprotein hSPAR functions as a glutamine inhibitor by promoting the translocation of accumulated P27KIP1 from the cytoplasm to lysosomes, where it subsequently prevents the assembly of the mTORC1 complex and its associated signalling. Importantly, these effects appear to be specific to BC cells, as they are not observed in HEK293T or non‐malignant MCF10A cells [37].

The Wnt/β‐catenin pathway is a crucial pathway for cancer cell proliferation and differentiation [51], which is often hyperactive in TNBC due to the accumulation and nuclear translocation of β‐catenin [52]. Targeting this pathway, the novel microprotein **miPEP205**, which is encoded by the lncRNA MIR205HG, has been identified and characterised as a tumour suppressor for TNBC [38]. Mechanistically, miPEP205 enhances phosphorylation of Glycogen Synthase Kinase 3 Beta at the Tyr216 residue, leading to cytoplasmic β‐catenin degradation and subsequent inhibition of malignant progression in TNBC tissues. Preclinical validation using the MMTV‐PyMT spontaneous BC murine model verified the therapeutic potential of miPEP205 [38]. Both genetic introduction and direct administration of miPEP205 significantly reduced primary tumour growth, attenuated lung metastasis, and improved survival in tumour‐bearing mice [38].

LINC00908 emerged as the first microprotein‐encoding lncRNA with differential expression in TNBC, identified by Wang et al. through the Cancer Genome Atlas database and Genome Wide Information on Protein Synthesis visualised dataset [39]. As confirmed by luciferase reporter assay, they showed LINC00908 expression and its encoded microprotein were directly downregulated by estrogen receptor alpha expression in TNBC lines (MDA‐MB‐231 and Hs578T). Subsequent investigations revealed that the resulting microprotein binds to signal transducer and activator of transcription 3 (STAT3) via its coiled‐coil domain, thereby reducing STAT3 phosphorylation and vascular endothelial growth factor (VEGF) expression [39]. This microprotein is considered a small regulatory peptide of STAT3 (**ASRPS**). In a TNBC mouse model, intravenous injection of ASRPS significantly reduced angiogenesis and tumour growth, exhibiting its promise as an anti‐angiogenic therapeutic in this aggressive subset of BC [39].

Despite the uncertain mechanism, some microproteins show an inhibitory effect on BC metastasis. MAGI2 antisense RNA 3 (MAGI2‐AS3) is a lncRNA with low expression in BC with anti‐tumour effects. By exploring its protein‐coding potential using TransLnc database, Zhang et al. identified five ORFs within MAGI2‐AS3, one of which encodes a microprotein named **MAGI2‐AS3‐ORF5** in MCF7 and MDA‐MB‐436 cells [40]. A Venn diagram analysis revealed 36 proteins interacting with MAGI2‐AS3‐ORF5, many of which were correlated with tumour size. Notably, four of these (COL18A1, COL12A1, HSPG2, and LAMC1) are extracellular matrix (ECM)‐associated proteins, which suggests that MAGI2‐AS3‐ORF5 may influence BC cell migration by modulating ECM‐related pathways, offering insight into its potential mechanism of action at the protein level [40]. At the RNA level, MAGI2‐AS3 has previously been shown to inhibit the Wnt/β‐catenin signalling pathway by upregulating MAGI2 [53]. Du et al. pointed out that MAGI2‐AS3 inhibits metastatic progression by acting as a molecular sponge for miR‐374a, resulting in elevated phosphatase and tensin homologue expression [54]. Additionally, Yang et al. reported that MAGI2‐AS3 is downregulated in BC and contributes to anti‐tumour activity by targeting the Fas‐FasL apoptotic pathway [55]. Collectively, these studies underscore the tumour‐suppressive role of MAGI2‐AS3 in BC. However, the exact contribution of MAGI2‐AS3‐ORF5 as a tumour‐suppressing microprotein in mediating these effects remains to be fully elucidated and requires further investigation.

Moreover, the transforming growth factor beta (TGF‐β) pathway has complex roles in BC, which can induce epithelial‐mesenchymal transition. Its inhibition has been shown to enhance chemotherapy efficacy in TNBC [56]. Studies have shown that TGF‐β treatment can directly influence lncRNA at the transcription level [57]. For example, Guo et al. identified a lncRNA LINC00665, that encodes a microprotein, named **CIP2A‐BP**, that is downregulated by TGF‐β treatment in TNBC mouse models. TGF‐β activates the Smad signalling pathway, which induces reduced translation of CIP2A‐BP from LINC00665 [18]. Downregulation of CIP2A‐BP is associated with tumour invasion and metastasis, as well as poor overall survival [18]. CIP2A‐BP functions as a tumour suppressor in TNBC by binding to and inhibiting CIP2A, a known tumour promoter in up to 90% of BCs [18]. Notably, in the MMTV‐PyMT mouse model, either upregulating CIP2A‐BP or directly administering the microprotein led to a significant reduction in lung metastases and improved survival outcomes [18].

### 3.3 Bidirectional Regulation Role of Microproteins

It is worth noting that LINC00511‐133aa, miPEP205, and MAGI2‐AS3‐ORF5 exert the contrasting roles in modulating the Wnt/β‐catenin pathway, which illustrates that the same signalling axis can be co‐opted in opposing directions depending on cellular context. LINC00511‐133aa activates Wnt/β‐catenin signalling to promote stemness [34], whereas miPEP205 suppresses the same pathway by enhancing GSK‐3β‐mediated cytoplasmic degradation of β‐catenin [38], and MAGI2‐AS3‐ORF5 is associated with Wnt/β‐catenin inhibition through upregulation of MAGI2 at the RNA level [40]. This bidirectionality is not paradoxical but rather reflects the well‐established context‐dependency of Wnt signalling in BC. LINC00511‐133aa operates within a cancer stem cell subpopulation, where Wnt activation is a physiologically conserved mechanism for self‐renewal [34], while miPEP205 acts in bulk tumour cells, where aberrant nuclear accumulation of β‐catenin drives uncontrolled proliferation [38]. The subcellular localisation of β‐catenin is therefore a critical determinant of pathway outcome, and its cytoplasmic degradation represents a tumour‐suppressive event, whereas its nuclear translocation drives oncogenic transcription of stemness and proliferative target genes. Whether the subcellular localisation of each microprotein itself contributes to this differential regulation remains an important open question. These findings collectively highlight the Wnt/β‐catenin pathway as a particularly dynamic node of microprotein‐mediated regulation in BC, and underscore the necessity of interpreting microprotein function within its specific cellular and molecular context.

## 4 Conceptual Clinical Applications and Future Perspectives

As discussed in this review, microproteins encoded from smORFs are pervasively involved in tumorigenesis and tumour progression by closely influencing key cellular processes such as proliferation, stress response, suppressive effects, apoptosis, and migration. The nascent field of microproteins holds transformative promise for BC management, such as the development of novel antineoplastic drugs and cancer biomarkers.

### 4.1 Microproteins as Targets/Agents for Therapy

**FIGURE 2:**
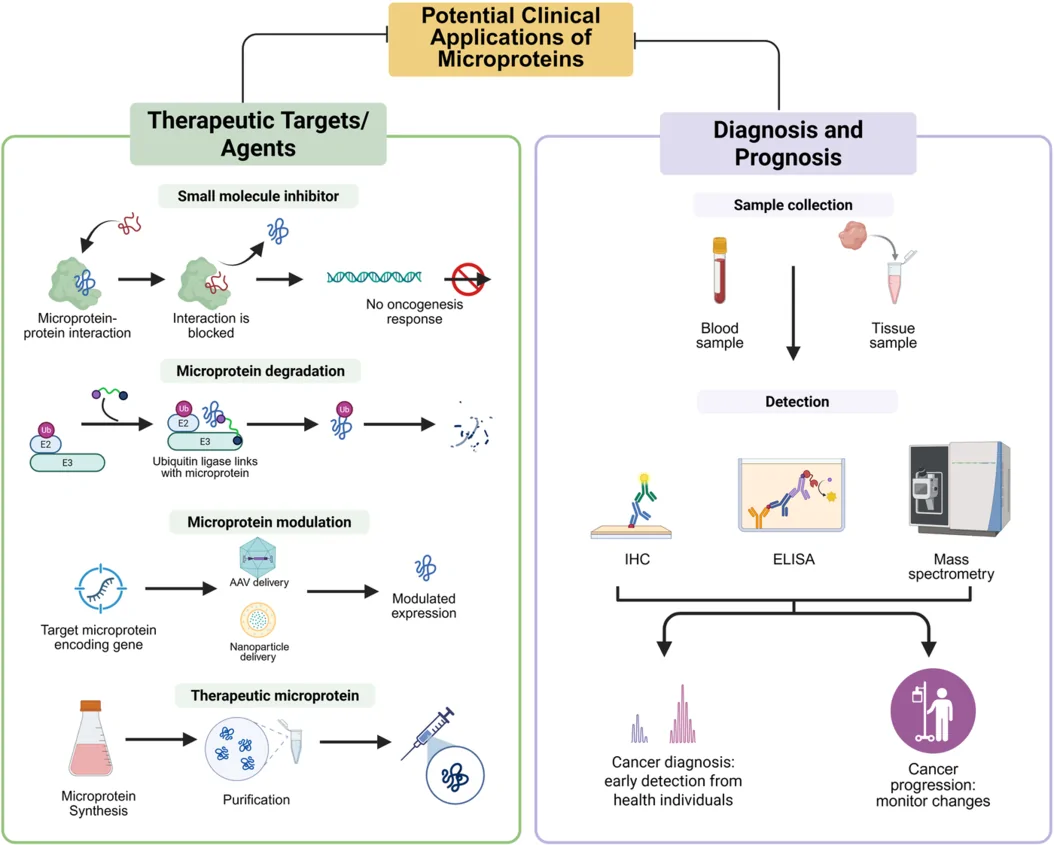
Potential therapeutic application of microprotein in BC management (created with BioRender.com). (Left panel, green) Potential therapies that target tumour‐promoting microproteins include small molecule inhibitors, proteolysis‐targeting chimeras, microprotein modulation, and therapeutic microproteins. Development of therapies could be guided using computational approaches. (Right panel, purple) Microproteins are emerging as potential biomarkers for the diagnosis and prognosis of BCs.

Evidence indicates that knockdown or inhibition of oncogenic microproteins contributes to reduced cancer cell proliferation and improved cancer prognosis [30] and also enhances the efficacy of conventional anticancer therapies [18]. Given that most microproteins function through protein–protein interactions (PPIs), a small molecule inhibitor could be developed to target and disrupt this interaction (Figure 2, left). As one of the most adaptable therapeutic modalities, small molecule inhibitors can be chemically tuned for efficient cellular membrane crossing, oral administration, and selective tissue distribution [58]. However, many microprotein exert their effects through protein scaffolding or structural roles rather than enzymatic activity. In such a context, proteolysis‐targeting chimeras (PROTACs) could be an alternative route by introducing degradation of oncogenic microproteins [59]. PROTACs are bifunctional molecules that consist of a ligand for the target protein (microprotein) linked to an E3 ubiquitin ligase‐recruiting element, forming a ternary complex that leads to the ubiquitination and proteasomal degradation of the target microprotein [60]. Of course, the success of both inhibition‐ and degradation‐based strategies hinges on several unresolved challenges. First, obtaining high‐resolution structures of relevant PPI complexes for the microprotein of interest remains a major bottleneck, as microproteins are still poorly characterised and structurally underdetermined. Moreover, identifying which microprotein‐PPI axes are truly rate‐limiting for cancer progression is essential to ensure meaningful therapeutic effects. Finally, achieving sufficient selectivity represents another critical hurdle to overcome to avoid unintended perturbation of function in normal tissue.

Alternatively, there is growing interest in modulating endogenous microprotein expression levels as a therapeutic strategy (Figure 2, left), which leverages gene‐ and RNA‐based technologies to upregulate tumour‐suppressive microproteins or silence oncogenic ones. This approach is supported by the inherently high specificity of microprotein–target interactions, which, in principle, could minimise off‐target toxicity compared to conventional small molecules. A range of platforms enable tunable control over microprotein expression levels, including adeno‐associated viral vectors with cell‐type specific promoters [61] and lipid nanoparticles [62].

However, despite these conceptual advantages, the indirect modulation of endogenous microprotein expression is burdened by complexity at both the sequence and systems levels. At the sequence level, antisense‐based approaches could easily suffer from imperfect specificity. Even minimal mismatches can result in unintended transcript suppression, leading to widespread off‐target effects. More critically, many microproteins are encoded within lncRNAs that possess multiple regulatory functions systematically. Their host transcripts frequently participate in chromatin organisation, transcriptional regulation, and competing endogenous RNA networks, meaning that their perturbation can propagate through extensive gene regulatory circuits. Further efforts and refinement are needed to achieve precise tissue‐specific delivery for safe clinical translation of this conceptual approach.

In contrast, the direct administration of microproteins offers a potentially more controllable strategy by bypassing transcriptional and post‐transcriptional regulation altogether. In this model, synthetic or recombinant microproteins are delivered exogenously to directly engage their intracellular targets, functioning as compact, highly specific modulators of protein–protein interactions or signalling pathways. Preclinical studies provide proof‐of‐concept for this approach: intratumoral administration of the tumour‐suppressive microprotein, ASRPS, has demonstrated the ability to inhibit angiogenesis through modulation of the STAT3/VEGF axis, which significantly prolonged survival in the TNBC mouse model [39].

Nevertheless, the translational viability of direct microprotein therapeutics remains constrained by several interdependent pharmacological and biophysical limitations. Since microproteins are vulnerable to proteolytic degradation in serum, extracellular fluid, and intracellular compartments, a highly specific and biocompatible delivery capsule would be needed to improve stability and half‐life of microproteins following administration. Another instability is contributed by microproteins' rapid clearance mechanism due to their small molecular size, which is typically below 10–12 kDa and well beneath the glomerular filtration threshold of 60–70 kDa. This renders them highly susceptible to rapid renal clearance via glomerular filtration, substantially reducing systemic bioavailability and the time available to reach tumour tissue [63]. Moreover, exogenously administered microproteins, particularly those derived from non‐endogenous sequences or delivered at supraphysiological concentrations, carry a clinical risk of immunogenicity. The adaptive immune system can recognise microprotein therapeutics as foreign antigens and mount anti‐drug antibody responses that neutralise therapeutic activity, alter pharmacokinetics, or, in severe cases, trigger adverse immune reactions [64, 65]. Taken together, advances in microprotein stabilisation, renal retention engineering, and immunogenicity mitigation will be required for the clinical translation of direct microprotein therapeutics.

### 4.2 Microproteins as Diagnostic and Prognostic Biomarkers

Compared to healthy cells and tissues, microproteins have shown distinct expression patterns in BC, which underscores their potential as biomarkers in early detection and helps determine follow‐up treatment according to prognosis (Figure 2, right). For example, the microprotein MRP, which is highly expressed in malignant BC cells, can differentiate patients with lymph node metastasis from those without, with an area under the curve of 0.7112 in the receiver operating assay, indicating its potential as a diagnostic biomarker in discriminating BC patients with or without lymph node metastasis [31]. Moreover, there have been several studies that have targeted ER stress as a therapeutic approach to cancer. Choi and Kang et al. demonstrated that bortezomib, a clinically available ER‐stress‐inducing anti‐cancer agent, exerts an enhanced effect in MTLN‐deficient BC cells [29]. Thus, the expression level of MTLN may serve as a plausible prognostic biomarker for predicting sensitivity to proteasome inhibitors or other ER‐stress‐inducing treatments [29]. Certain microproteins have been linked to tumour aggressiveness and survival rates. TNBC‐specific microproteins, ASRPS and HCP5‐132aa, have demonstrated a positive correlation with poor survival rate [35, 39], suggesting their potential as indicators of aggressive tumour behaviour and treatment response.

### 4.3 Future Challenges

Despite the promising clinical potential of microproteins as therapeutic agents, there are still many challenges that must be addressed to translate microprotein research from the bench to the bedside. To date, only a few BC‐specific microproteins have been characterised, while many more likely await discovery and validation as therapeutic molecules in the near future. Yet, detecting these microproteins remains inherently challenging due to their small size and low abundance in biological samples. Standard proteomic techniques either lack the necessary sensitivity or require complicated preparation protocols for reliable detection. Moreover, the absence of well‐annotated sequences further complicates functional validation. Therefore, the development of enhanced mass spectrometry protocols and bioinformatic tools for annotation is crucial for overcoming these obstacles. In parallel, developing robust assays and model systems that accurately recapitulate the complexity of the cancer phenotype is essential for validating microprotein function in both experimental and clinical settings. Furthermore, although some microproteins have exhibited cell‐ and tissue‐specific expression patterns, it is imperative to precisely map their distribution across different tissues to ensure reliable sample sourcing and avoid false positives. Additionally, targeting specific microproteins may unintentionally disrupt other cellular processes. A thorough understanding of their upstream regulatory mechanisms and downstream binding partners is necessary to minimise off‐target effects of microproteins, especially for systematic therapeutic usage.

## 5 Conclusion

Advances in transcriptomics and genomics over the past decade have transformed our understanding of ncRNA, elevating them from “transcriptional noise” to potential functioning entities capable of encoding microproteins that regulate cell fate and homeostasis. This shift has revealed a new complex landscape involving both physiological and pathophysiological processes in many cells, including BC. Recent studies have identified several microproteins as key players in BC progression, influencing tumour proliferation, migration, and resistance to chemotherapy. This review provides an overview of the mechanistic roles of these microproteins, highlights state‐of‐the‐art discovery methods, and discusses currently characterised functional microproteins in BC, while also considering their potential for future clinical application. Despite these promising findings, the field remains in its early stages. The discovery and functional annotation of novel microproteins are ongoing with a constant need for novel treatment strategies, especially for TNBC, which lacks reliable clinical biomarkers and targeted therapies, resulting in a poorer prognosis characterised by higher nuclear grades [66].

In conclusion, microproteins represent a promising frontier in the diagnosis, prognosis, and treatment of BC. With the continued development of advanced tools, such as high‐throughput screening and improved delivery technologies, the biological functions and modulating mechanisms of microproteins may soon be fully understood, unlocking new strategies for personalised BC management.

## Disclosure

This review reflects the authors' interpretation of the currently available literature. The opinions and conclusions expressed are solely those of the authors and do not necessarily represent the views of their affiliated institutions or the publisher.

## Conflicts of Interest

The authors declare no conflicts of interest.

## Data Availability

Data sharing is not applicable to this article, as no new data were created or analysed in this study.
